# Clinical phenotyping of spondylodiscitis and isolated spinal epidural empyema: a 20-year experience and cohort study

**DOI:** 10.3389/fsurg.2023.1200432

**Published:** 2023-05-18

**Authors:** Mido Max Hijazi, Timo Siepmann, Ibrahim El-Battrawy, Patrick Glatte, Ilker Eyüpoglu, Gabriele Schackert, Tareq A. Juratli, Dino Podlesek

**Affiliations:** ^1^ Department of Neurosurgery, University Hospital Carl Gustav Carus, Technische Universität Dresden, Dresden, Germany; ^2^ Department of Neurology, University Hospital Carl Gustav Carus, Technische Universität Dresden, Dresden, Germany; ^3^ Department of Cardiology, Bergmannsheil University Hospitals Bergmannsheil, Ruhr University Bochum, Bochum, Germany

**Keywords:** spondylodiscitis, vertebral ostemyelitis, sepsis, endocarditis, septic embolism, primary spinal infection, isolated spinal epidural empyema

## Abstract

**Background:**

The incidence of spondylodiscitis (SD) and isolated spinal epidural empyema (ISEE) has been increasing in the last decades, but the distinct differences between both entities are poorly understood. We aimed to evaluate the clinical phenotypes and long-term outcomes of SD and ISEE in depth.

**Methods:**

We performed a chart review and analyzed data from our cohorts of consecutive SD and ISEE patients who were treated and assessed in detail for demographic, clinical, imaging, laboratory, and microbiologic characteristics at a university neurosurgical center in Germany from 2002 to 2021. Between-group comparisons were performed to identify meaningful differences in both entities.

**Results:**

We included 208 patients (72 females: age 75 [75 32–90] y vs. 136 males: 65 [23–87] y, median [interquartile range], *p* < 0.001), of which 142 (68.3%) had SD and 66 (31.7%) had ISEE. Patients with SD were older than ISEE (ISEE: 62 y vs. SD: 70 y, *p* = 0.001). While SD was more common in males than females (males: *n* = 101, 71.1% vs. females: *n* = 41, 28.9%, *p* < 0.001), there was no sex-related difference in ISEE (males: *n* = 35, 53.0% vs. females: *n* = 31, 47.0%, *p* = 0.71). Obesity was more frequent in ISEE than in SD (ISEE: *n* = 29, 43.9% vs. SD: *n* = 37, 26.1%, *p* = 0.016). However, there were no between-group differences in rates of diabetes and immunodeficiency. In the entire study population, a causative pathogen was identified in 192 (92.3%) patients, with methicillin-susceptible staphylococcus aureus being most frequent (*n* = 100, 52.1%) and being more frequent in ISEE than SD (ISEE: *n* = 43, 65.2% vs. SD: *n* = 57, 40.1%, *p* = 0.003). SD and ISEE occurred most frequently in the lumbar spine, with no between-group differences (ISEE: *n* = 25, 37.9% vs. SD: *n* = 65, 45.8%, *p* = 0.297). Primary infectious sources were identified in 145 patients (69.7%) and among this skin infection was most common in both entities (ISEE: *n* = 14, 31.8% vs. SD: *n* = 25, 24.8%, *p* = 0.418). Furthermore, epidural administration was more frequent the primary cause of infection in ISEE than SD (ISEE: *n* = 12, 27.3% vs. SD: *n* = 5, 4.9%, *p* < 0.001). The most common surgical procedure in SD was instrumentation (*n* = 87, 61%) and in ISEE abscess evacuation (*n* = 63, 95%). Patients with ISEE displayed lower in-hospital complication rates compared to SD for sepsis (ISEE: *n* = 12, 18.2% vs. SD: *n* = 94, 66.2%, *p* < 0.001), septic embolism (ISEE: *n* = 4/48 cases, 8.3% vs. SD: *n* = 52/117 cases, 44.4%, *p* < 0.001), endocarditis (ISEE: *n* = 1/52 cases, 1.9% vs. SD: *n* = 23/125 cases, 18.4%, *p* = 0.003), relapse rate (ISEE: *n* = 4/46, 8.7% vs. SD: *n* = 27/92, 29.3%, *p* = 0.004), and disease-related mortality (ISEE: *n* = 1, 1.5% vs. SD: *n* = 11, 7.7%, *p* = 0.108). Patients with SD showed prolonged length of hospital stay (ISEE: 22 [15, 30] d vs. SD: 38 [29, 53] d, *p* < 0.001) and extended intensive care unit stay (ISEE: 0 [0, 4] d vs. SD: 3 [0, 12] d, *p* < 0.002).

**Conclusions:**

Our 20-year experience and cohort analysis on the clinical management of SD and ISEE unveiled distinct clinical phenotypes and outcomes in both entities, with ISEE displaying a more favorable disease course with respect to complications and relapse rates as well as disease-related mortality.

## 1. Introduction

Spondylodiscitis (SD) affects the vertebral body, intervertebral disc, and/or adjacent paraspinal tissues, due to an adjacent source (trauma, surgery, intervention) or hematogenous spread (1–3). However, isolated spinal epidural empyema (ISEE) is an infection of the epidural space with accumulation of purulent substance in the cavity between the dura and the osseo-ligamentous boundaries of the spinal canal, without findings of spondylodiscitis on magnetic resonance imaging (MRI) or intraoperatively (4). On the other hand, epidural empyema/abscess can be a secondary complication of a primary SD, spreading hematogeneously via septic thrombosis of epidural veins (5).

The incidence of SD and ISEE has been increasing over the last decades, due to an aging population with serious comorbidities and increasing spinal interventions, advanced imaging, and a growing number of drug abusers (6, 7). It ranges from 1:20,000 to 1:100,000, and the mortality rate varies between 2% and 20% in industrialized countries (2, 8–11). Diabetes mellitus, immunosuppression, and human immunodeficiency virus (HIV) infection are additional risk factors associated with SD and ISEE (12).

SD is commonly (55%–80%) caused by methicillin-sensitive staphylococcus aureus (MSSA) and spreads hematogeneously (13). MSSA is also the most common responsible pathogen in ISEE (14–17). Primary SD is associated with a more severe course and significantly higher mortality rate than secondary acquired SD, which is triggered by postoperative infections (18). The majority of ISEE occurs iatrogenically, following spinal surgical procedures, epidural anaesthesia, or spinal injections (19).

Although both pathologies represent clinically distinct etiologic and anatomic entities that require different treatments, only a few studies have addressed detailed differences between SD and ISEE. Therefore, here we aimed to evaluate and compare the clinical features, complications, treatments, and outcomes of both pathologic entities over 20 years.

## 2. Materials and methods

### 2.1. Study design

We performed a retrospective observational study of consecutive patients with SD or ISEE who underwent surgical treatment from 2002 to 2021 at our university neurosurgical spine centre in Dresden/Germany. Patients with late or early postoperative spinal infections were not considered in this study as the focus was on patients with primary spinal infections and not on secondary spinal infections resulting after surgery.

All patients who underwent surgery and had no intradural infection on admission between 2002 and 2021 were included. Twenty patients were excluded due to one of the following criteria:

- only conservative treatment
- intradural infection (subdural abscess or spinal cord abscess)

**Figure 1:**
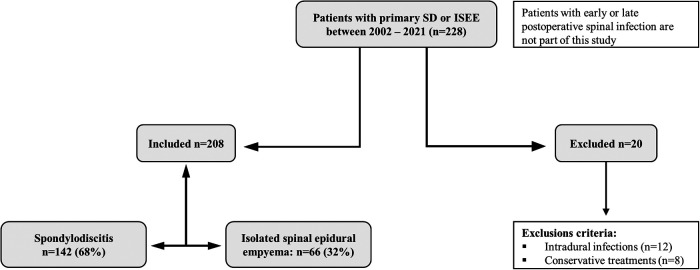
Study design. Study design: Two hundred twenty-eight patients had spondylodiscitis (SD: 142) or isolated spinal epidural empyema (ISEE: 66) without early or late postoperative spinal infection. Twenty patients were excluded due to exclusion criteria.

Two hundred twenty-eight patients with SD and ISEE were identified, whereas SD was diagnosed in 142 patients (68.3%) and ISEE in 66 patients (31.7%) (Figure 1).

### 2.2. Patient data

The study was approved by our local ethics committee (Reference number BO-EK-17012022). After case identification, patient data were extracted by reviewing electronic medical records using the ORBIS system (ORBIS, Dedalus, Bonn, Germany). Radiological data, including gadolinium contrast-enhancing MRI, computer tomography (CT) and/or x-ray were available for review in all cases. Collected data included demographic information, risk factors, causative pathogens, spinal localization of infection, primary infectious source, surgical and antibiotic treatment, type of surgical procedure, length of hospital and intensive care stay, and complications such as sepsis, septic embolisms, endocarditis, relapse rates, and disease-related mortality.

### 2.3. Clinical management

SD or ISEE were diagnosed based on clinical history, fever, leukocyte count, C-reactive protein (CrP), typical radiological changes on MRI, and pathogen detection in blood cultures, intraoperative specimens, or CT-guided biopsies (20). Transthoracic echocardiogram (TTE) was performed in all patients, but transoesophageal echocardiogram (TEE) was obtained in patients with suspected endocarditis according to the modified DUKE criteria (21). First-line treatment was usually conservative with intravenous antibiotics, although surgical treatment was indicated in cases of source control, epidural abscess, neurologic deficits, or spinal instability. The type of surgical intervention was determined in the interdisciplinary spine conference or the neurosurgical-neuroradiological conference depending on the clinical, laboratory, and radiological findings. Between 2002 and 2015, cases were discussed in our neurosurgical-neuroradiology conference and treated in collaboration with infectious disease physicians. Since 2015, a multidisciplinary spine conference has been conducted involving neuroradiologists, neurosurgeons, trauma surgeons, orthopaedic surgeons, and infectious disease physicians to determine a treatment strategy for patients. Patients with ISEE underwent abscess evacuation with/without drainage or anterior cervical discectomy and fusion (ACDF) for abscess ventral to the spinal cord in the cervical spine, whereas patients with SD were treated with either abscess evacuation or instrumentation in cases of instability, deformity, and pain-related immobility. Therefore, surgical decision making depends on clinical experience and various defined radiological features. Preoperative CT scans were performed in all patients undergoing spinal instrumentation to assess bony integrity.

All patients received either a targeted antibiotic or empirical antibiotic treatment depending on the clinical condition at admission and following the recommendations of the local department of infectious diseases. The empirical antibiotic therapy was switched to targeted after detection of the causative pathogens. In SD patients, intravenous antibiotic therapy was switched to oral antibiotics after approximately 4–6 weeks, and the total duration of antibiotic therapy ranged from 10 to 12 weeks. On the other hand, ISEE patients received a 2-weeks intravenous antibiotic treatment, which was changed to oral administration with a total duration that ranged from 4 to 6 weeks. Clinical and radiological follow-up was performed on all patients who complied with our recommendation at 3, 6, and 12 months after hospital discharge.

### 2.4. Statistical analysis

All statistical analyses were carried out using the software package SPSS (SPSS Statistics 28, IBM, Armonk, New York, USA). Descriptive statistics were used, and categorical variables were compared between ISEE and SD using Fisher's exact tests when appropriate or Chi-squared test. Numeric variables were compared using Mann–Whitney *U*-tests when appropriate. A binomial test was used to analyse the distribution of gender. All statistical tests were two-tailed, and a *p*-value *p* < 0.05 was considered statistically significant. Univariate and multivariate analyses were performed to identify independent risk factors.

## 3. Results

### 3.1. Clinical characteristics

#### 3.1.1. Demographic distribution

We enrolled 136 male patients (65.4%) and 72 female patients (34.6%), with males being significantly younger than females at the time of diagnosis (males: 65 y vs. females: 75 y, *p* < 0.001). SD patients were significantly older than ISEE (ISEE: 62 y vs. SD: 70 y, *p* = 0.001). In addition, SD occurred predominantly in male patients (males: 71.1% vs. females: 28.9%, *p* < 0.001), and male patients with SD were younger than females at the time of diagnosis (males: 67 y vs. females: 76 y, *p* < 0.001). In ISEE, sex (males: 53% vs. females: 47%, *p* = 0.712) and age (males: 60 y vs. females: 69 y, *p* = 0.063) were equally distributed (Table 1).

**Table 1:** Demographic distribution of SD and ISEE.

Age/Gender	SD & ISEE (*n* = 208, 100%)	SD (*n* = 142, 68.3%)	ISEE (*n* = 66, 31.7%)	*p*-value
Males	136 (65.4%)	101 (71.1%)	35 (53.0%)	**0**.013^**^
Females	72 (34.6%)	41 (28.9%)	31 (47.0%)	
*p*-value	**<0.001** ^*^	**<0.001** ^*^	0.712^*^	
Age in years (median)	68 (23–90)	70 (38–90)	62 (23–83)	**0**.001^***^
Age of males (median)	65 (23–87)	67 (38–87)	60 (23–83)	**0**.007^***^
Age of females (median)	75 (32–90)	76 (42–90)	69 (32–83)	**0**.002^***^
*p*-value	**0.001** ^***^	**<0.001** ^***^	0.063^***^	

SD, spondylodiscitis; ISEE, isolated spinal epidural empyema.

* Binomial test

** Fisher's exact test

*** Mann–Whitney *U*-tests.

Bold values are significant results (*p* < 0.05) as indicated in the methods.

#### 3.1.2. Risk factors

Age and sex are counted among the risk factors and are presented in the upper paragraph. Diabetes mellitus was observed in 77 patients (37%) and showed no difference between both groups (ISEE: *n* = 18, 27.3% vs. SD: *n* = 59, 41.5%, *p* = 0.064). A total of 66 patients with BMI (body mass index) higher than 30 kg/m^2^ (31.7%) were identified, whereas obesity was more frequent in ISEE than in SD (ISEE: *n* = 29, 43.9% vs. SD: *n* = 37, 26.1%, *p* = 0.016). We found 35 immunocompromised patients (16.8%) with no differences between the two entities (ISEE: *n* = 7, 10.6% vs. SD: *n* = 28, 19.7%, *p* = 0.115) (Table 2).

**Table 2:** Risk factors in patients with SD and ISEE.

Risk factors	SD & ISEE (*n* = 208, 100%)	SD (*n* = 142, 68.3%)	ISEE (*n* = 66, 31.7%)	*p*-value
Diabetes mellitus	77 (37.0%)/131 (63.0%)	59 (41.5%)/83 (58.5%)	18 (27.3%)/48 (72.7%)	0.064^*^
Obesity (BMI > 30 kg/m^2^)	66 (31.7%)/142 (68.3%)	37 (26.1%)/105 (73.9%)	29 (43.9%)/37 (56.1%)	**0**.016^*^
Immunosuppressed patients	35 (16.8%)/173 (83.2%)	28 (19.7%)/114 (80.3%)	7 (10.6%)/59 (89.4%)	0.115^*^

SD, spondylodiscitis; ISEE, isolated spinal epidural empyema; BMI, body mass index.

*Fisher's exact test.

Bold values are significant results (*p* < 0.05) as indicated in the methods.

#### 3.1.3. Causative pathogens

**Figure 2:**
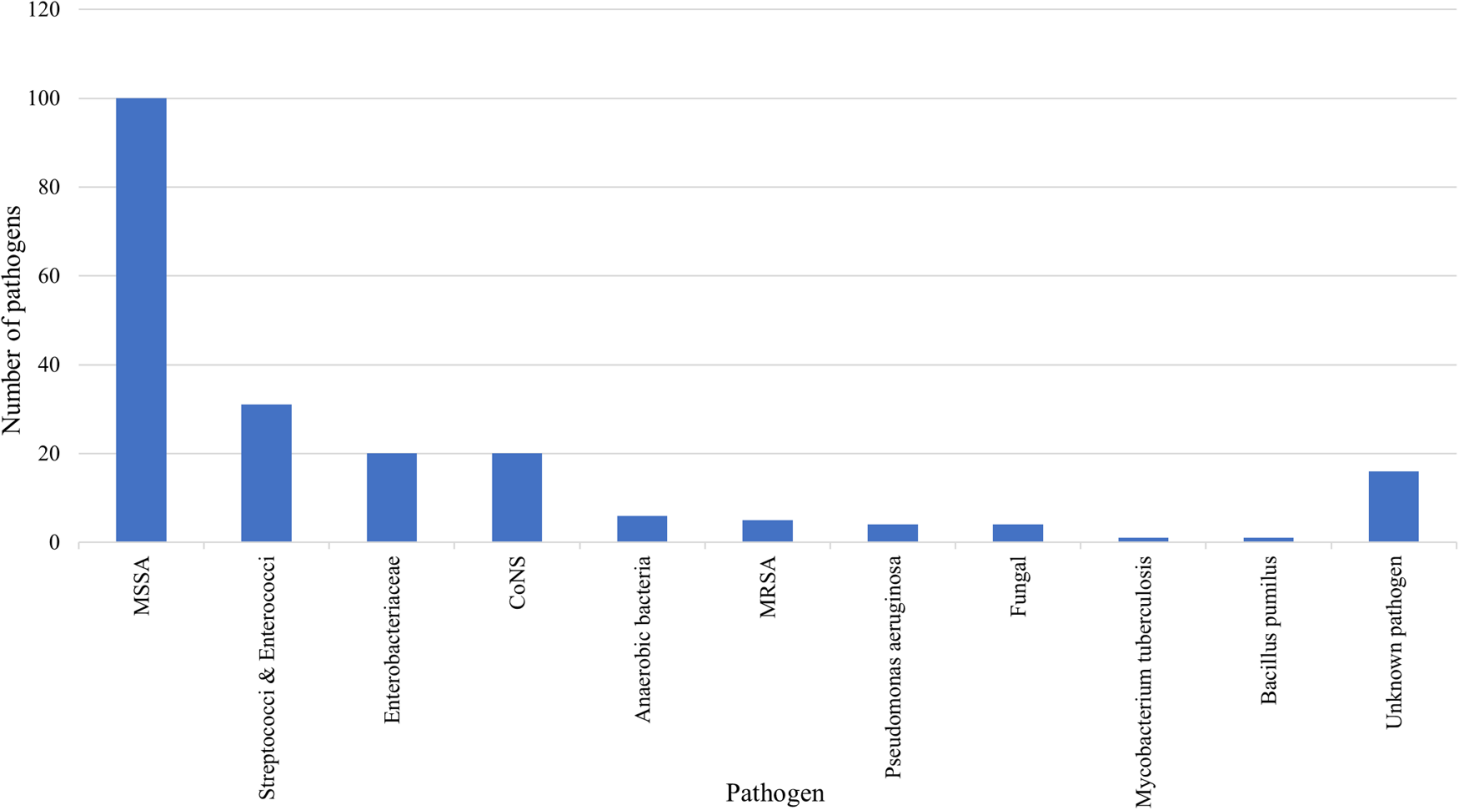
Causative pathogens of SD and ISEE. This figure presents the pathogens identified in spondylodiscitis (SD) and isolated spinal epidural empyema (ISEE). Methicillin-susceptible Staphylococcus aureus (MSSA): *n* = 100, 52.1%; streptococci and enterococci: *n* = 31, 16.1%; enterobacteriaceae: *n* = 20, 10.4%; coagulase-negative staphylococci (CoNS): *n* = 20, 10.4%; anaerobic bacteria: *n* = 6, 3.1%; methicillin-resistant staphylococcus aureus (MRSA): *n* = 5, 2.6%; pseudomonas aeruginosa: *n* = 4, 2.1%; fungi: *n* = 4, 2.1%; mycobacterium tuberculosis: *n* = 1, 0.5%; bacillus pumilus: *n* = 1, 0.5%.

A total of 192 pathogens (92.3%) were identified by blood cultures, intraoperative specimens, and/or CT-guided biopsies. In 16 patients (7.8%), the causative pathogens could not be isolated, presumably due to prior empiric antibiotic treatment. MSSA was the most common pathogen in both groups together (*n* = 100, 52.1%), followed by streptococci and enterococci (*n* = 31, 16.1%), enterobacteriaceae (*n* = 20, 10.4%), coagulase-negative staphylococci (*n* = 20, 10.4%), anaerobic bacteria (*n* = 6, 3.1%), methicillin-resistant staphylococcus aureus (*n* = 5, 2.6%), pseudomonas aeruginosa (*n* = 4, 2.1%), fungi (*n* = 4, 2.1%), mycobacterium tuberculosis (*n* = 1, 0.5%), and bacillus pumilus (*n* = 1, 0.5%) (Figure 2). MSSA was more common in ISEE than SD (ISEE: *n* = 43, 65.2% vs. SD: *n* = 57, 40.1%, *p* = 0.003).

#### 3.1.4. Localization in the vertebral column

The lumbar spine (LS) was the most affected part of the vertebral column (VC) in both entities (ISEE: *n* = 41, 62.1% vs. SD: *n* = 96, 67.6%). The presence in the LS alone was not significantly different between both groups (ISEE: *n* = 25, 37.9% vs. SD: *n* = 65, 45.8%, *p* = 0.297). The thoracic spine (TS) was less affected than the LS in both entities (ISEE: *n* = 33, 50% vs. SD: *n* = 49, 34.5%) and manifestation in the TS alone was also not significant between both groups (ISEE: *n* = 13, 19.7% vs. SD: *n* = 20, 14.1%, *p* = 0.314). The cervical spine (CS) was least affected in both entities (ISEE: *n* = 22, 33.3% vs. SD: *n* = 39, 27.5%), and infection appeared in the CS alone without significant difference between the two groups (ISEE: *n* = 7, 10.6% vs. SD: *n* = 21, 14.8%, *p* = 0.515). Infection was noted more frequently in ISEE than in SD in all three spinal segments combined (CS, TS, and LS). (ISEE: *n* = 9, 13.6% vs. SD: *n* = 6, 4.2%, *p* = 0.021) (Table 3).

**Table 3:** Occurrence rates of SD and ISEE in spinal segments.

Localization	SD (*n* = 142)	ISEE (*n* = 66)	*p*-value
CS	21 (14.8%)	7 (10.6%)	0.515^*^
TS	20 (14.1%)	13 (19.7%)	0.314^*^
LS	65 (45.8%)	25 (37.9%)	0.297^*^
CS & TS	5 (3.5%)	5 (7.6%)	0.294^*^
CS & LS	7 (4.9%)	1 (1.5%)	0.440^*^
TS & LS	18 (12.7%)	6 (9.1%)	0.641^*^
Complete vertebrae	6 (4.2%)	9 (13.6%)	**0**.021^*^

SD, spondylodiscitis; ISEE, isolated spinal epidural empyema; LS, lumbar spine; TS, thoracic spine; CS, cervical spine. *Fisher's exact test.

Bold values are significant results (*p* < 0.05) as indicated in the methods.

#### 3.1.5. Primary infectious source

If primary spinal infection is present as SD or ISEE, a primary infectious source can usually be identified. Infections that resulted directly from surgical spinal procedures were not addressed in this study. Primary infectious sources were identified in 145 patients (69.7%). Skin infections were the most common cause in both groups, with no significant difference between groups (ISEE: *n* = 14, 32.8% vs. SD: *n* = 25, 24.8%, *p* = 0.418). Furthermore, epidural administration was significantly more common in the ISEE group than in the SD (ISEE: *n* = 12, 27.3% vs. SD: *n* = 5, 5%, *p* < 0.001). However, no significant difference was found between both groups in respiratory tract infections (ISEE: *n* = 3, 6.8% vs. SD: *n* = 18, 17.8%, *p* = 0.125), gastrointestinal tract infections (ISEE: *n* = 0, 0.0% vs. SD: *n* = 9, 8.9%, *p* = 0.059), urinary tract infections (ISEE: *n* = 6, 13.6% vs. SD: *n* = 8, 7.9%, *p* = 0.356), port-associated infections (ISEE: *n* = 0, 0.0% vs. SD: *n* = 9, 8.9%, *p* = 0.059), retropharyngeal and prevertebral infections (ISEE: *n* = 1, 2.3% vs. SD: *n* = 7, 6.9%, *p* = 0.437), foreign body-associated infections like Hip-total endoprosthesis (ISEE: *n* = 2, 4.5% vs. SD: *n* = 6, 5.9%, *p* = 1.0), late infection associated with spinal screw elsewhere (ISEE: *n* = 0, 0.0% vs. SD: *n* = 6, 5.9%, *p* = 0.180), prosthetic valve endocarditis (ISEE: *n* = 0, 0.0% vs. SD: *n* = 3, 3.0%, *p* = 0.555), odontogenic infections (ISEE: *n* = 3, 6.8% vs. SD: *n* = 2, 2.0%, *p* = 0.155), immunodeficiency (ISEE: *n* = 0, 0.0% vs. SD: *n* = 2, 2.0%, *p* = 1.0). Root causes of infection remained undetermined in 63 patients (ISEE: *n* = 22, 33.3% vs. SD: *n* = 41, 28.9%) (Table 4).

**Table 4:** Primary infectious sources of SD and ISEE.

Primary infectious source	SD (*n* = 101/142)	ISEE (*n* = 44/66)	*p*-value
Skin infection	25 (24.8%)	14 (31.8%)	0.418^*^
Epidural administration	6 (5.9%)	15 (34.1%)	**<0**.001^*^
Respiratory tract infection	18 (17.8%)	3 (6.8%)	0.125^*^
Gastrointestinal tract infection	9 (8.9%)	0 (0.0%)	0.059^*^
Urinary tract infection	8 (7.9%)	6 (13.6%)	0.356^*^
Port associated infection	9 (8.9%)	0 (0.0%)	0.059^*^
Retropharyngeal and prevertebral space infection	7 (6.9%)	1 (2.3%)	0.437^*^
Foreign body associated infection like Hip-TEP	6 (5.9%)	2 (4.5%)	1.0^*^
Late infection associated with spinal screw elsewhere	6 (5.9%)	0 (0.0%)	0.180^*^
Prosthetic valve endocarditis	3 (3.0%)	0 (0.0%)	0.555^*^
Odontogenic infection	2 (2.0%)	3 (6.8%)	0.155^*^
Immunodeficiency	2 (2.0%)	0 (0.0%)	1.0^*^

SD, spondylodiscitis; ISEE, isolated spinal epidural empyema; primary infectious source was found in 145 patients (75%).

TEP, total endoprosthesis. *Fisher's exact test.

Bold values are significant results (*p* < 0.05) as indicated in the methods.

### 3.2. Treatment

#### 3.2.1. Surgical procedures

**Figure 3:**
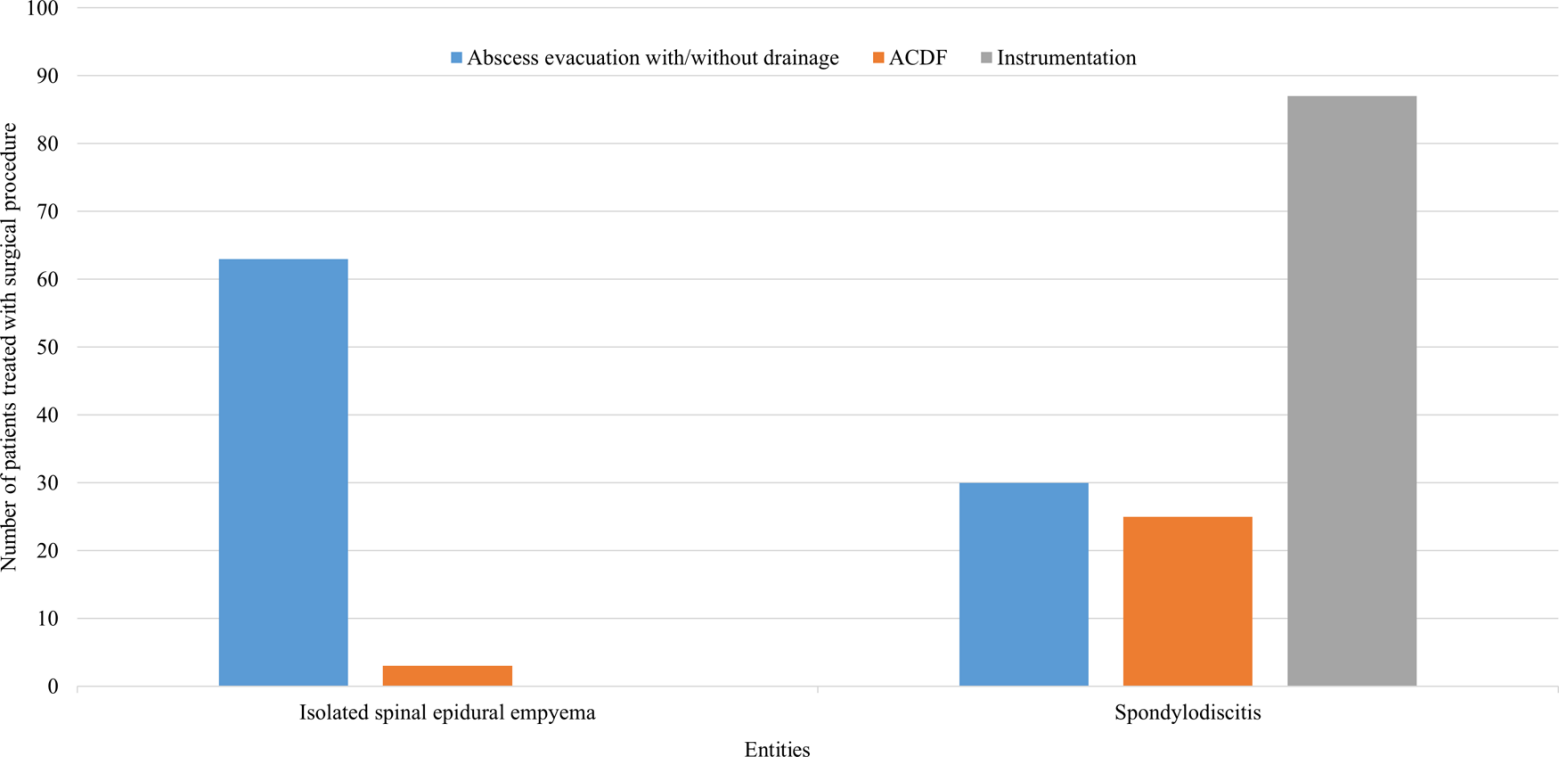
Surgical procedures used in SD and ISEE. This figure represents the surgical procedures for spondylodiscitis (SD) and isolated spinal epidural empyema (ISEE). ISEE were mainly addressed using abscess evacuation with/without drainage (*n* = 63, 95%), whereas SD were mostly managed with ventral, dorsal, or ventrodorsal instrumentation (*n* = 87, 61%). ACDF, anterior cervical discectomy with fusion.

Patients with ISEE received abscess evacuation with/without drainage in 95% (63 cases), whereas in only 5% (3 cases) abscess evacuation was managed by ACDF. No instrumentation was performed in any patient with ISEE. In contrast, instrumentation (ventral, dorsal, or ventrodorsal) was performed in 61% of SD (87 patients), whereas only 30 patients (21%) underwent abscess evacuation with/without drainage and 25 patients (18%) received ACDF (Figure 3).

#### 3.2.2. CT-guided biopsy in case of psoas abscess

A psoas abscess was found in 128 patients (61.5%), whereas 33.7% (70/208) of our cohort (SD and ISEE) underwent CT-guided biopsy. Psoas abscess was detected in 66.9% of SD patients (95/142), and 35.2% (50/142) of those underwent CT-guided biopsy with abscess drainage. Half (33/66) of the ISEE patients had developed a psoas abscess, and CT-guided biopsy with abscess drainage was performed in 30.0% (20/66).

#### 3.2.3. Antibiotic treatment

A total of 140 patients (67.3%) received empiric antibiotic therapy (ISEE: *n* = 51, 77.3% vs. SD: *n* = 89, 62.7%), which was switched to targeted antibiotic therapy based on pathogen identification. A causative pathogen was isolated in 68 patients (32.7%) before targeted antibiotic therapy began (ISEE: *n* = 15, 22.7% vs. SD: *n* = 53, 37.3%). Intravenous antibiotic therapy was switched to oral antibiotics in SD patients after 4 weeks (range: 0–19), and the total duration of antibiotic therapy was 10 weeks (range: 0–52). By contrast, ISEE patients received 2 week (range: 1–3) intravenous antibiotic treatment, which was then switched to oral administration for a total duration of 6 weeks (rage: 2–15).

### 3.3. Complications

**Figure 4:**
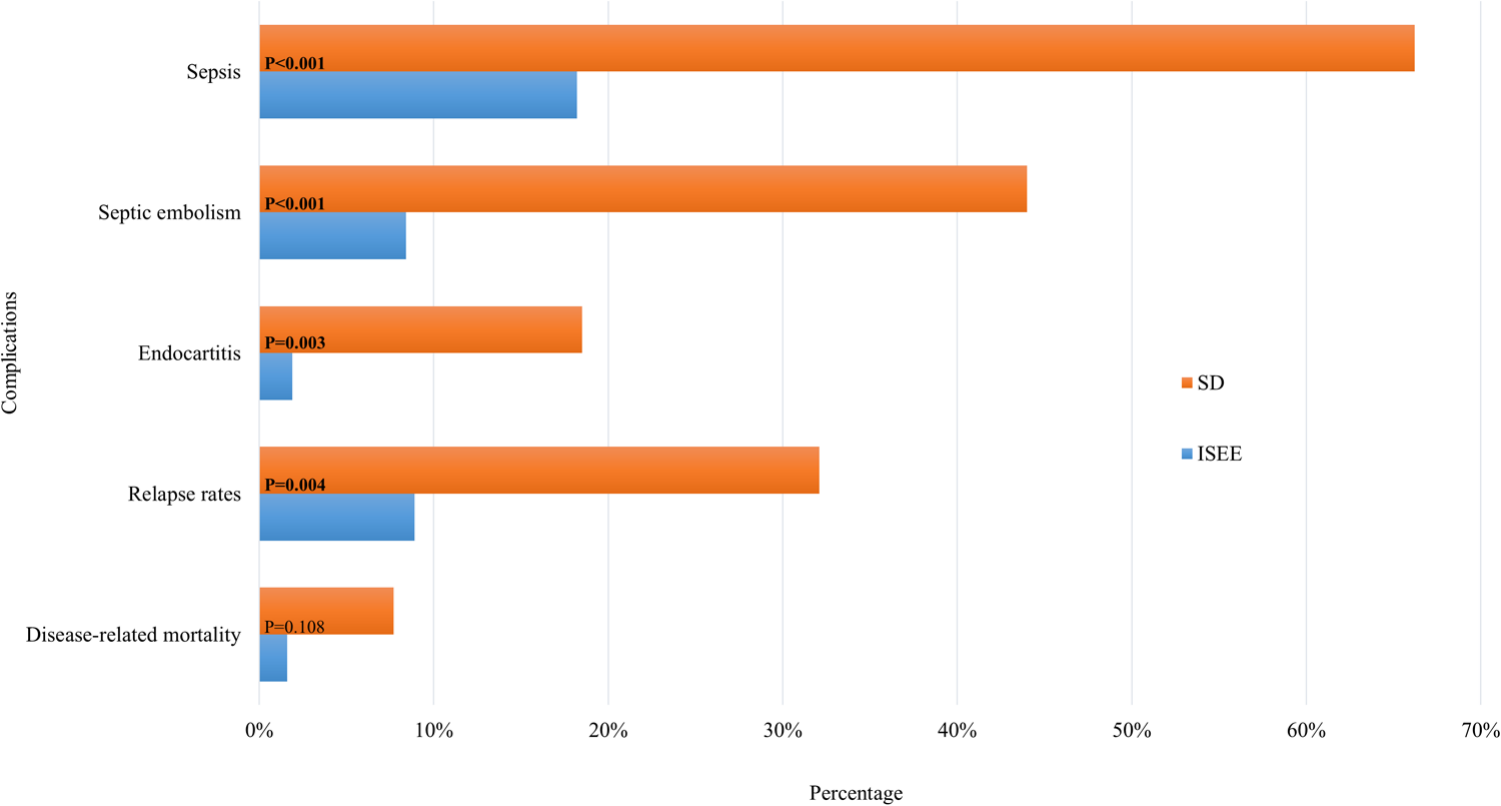
Complications in SD and ISEE. This figure shows the complications of spondylodiscitis (SD) and isolated spinal epidural empyema (ISEE). Sepsis, septic embolism, endocarditis, relapse rate and mortality rate demonstrate the contrast between the two pathologies. Required examinations to exclude septic embolism during disease course were lacking in 18 ISEE-patients and 25 SD-patients. TEE was not performed in 14 ISEE-patients and in 17 SD-patients. Relapse was defined as clinical, microbiological, and/or radiological progression during or after treatment up to the final follow-up of one year. Of note, 70 patients (50 SD, 20 ISEE, 33.7%) were lost to follow-up. * Indicates *p*-value in fisher's exact test.

Patients with ISEE were less likely to develop sepsis during the disease course than patients with SD (ISEE: *n* = 12, 18.2% vs. SD: *n* = 94, 66.2%, *p* < 0.001). Septic embolism occurred similarly less in ISEE than SD (ISEE: *n* = 4/48 cases, 8.3% vs. SD: *n* = 52/117 cases, 44.4%, *p* < 0.001). Required examinations to exclude septic embolism during disease course were lacking in 18 ISEE-patients and 25 SD-patients. We identified less infective endocarditis with proven vegetation in TTE in ISEE than in SD (ISEE: *n* = 1/52 cases, 1.9% vs. SD: *n* = 23/125 cases, 18.4%, *p* = 0.003). TEE was not performed in 14 ISEE-patients and in 17 SD-patients. The relapse rate was higher in SD-patients (ISEE: *n* = 4/46, 8.7% vs. SD: *n* = 27/92, 29.3%, *p* = 0.004). Relapse was defined as clinical, microbiological, and/or radiological progression during or after treatment up to the final follow-up of one year. Of note, 70 patients (50 SD, 20 ISEE, 33.7%) were lost to follow-up. Disease-related mortality was not significantly different between both entities (ISEE: *n* = 1, 1.5% vs. SD: *n* = 11, 7.7%, *p* = 0.108) (Figure 4).

### 3.4. Length of stay in hospital and intensive care unit

**Figure 5:**
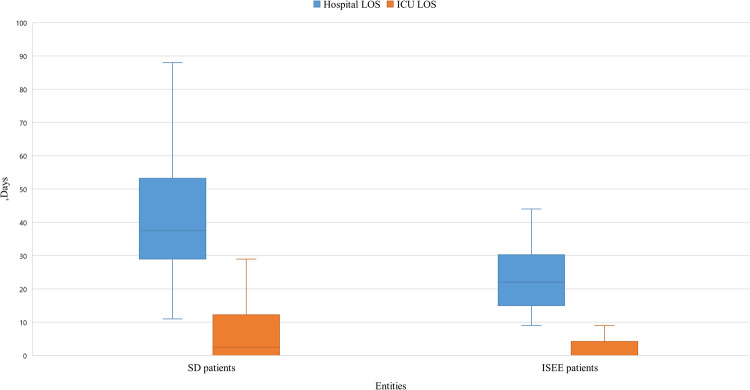
Length of stay in hospital and ICU of SD and ISEE. This figure displays the length of stay (LOS) in hospital and on the intensive care unit (ICU) in isolated spinal epidural empyema (ISEE) and spondylodiscitis (SD), which was significantly longer in the SD cohort than in the ISEE cohort (ISEE: 22 d vs. SD: 37 d, *p* < 0.001). LOS on the ICU differed significantly for both groups (median: ISEE 0 d vs. SD 2.5 d, *p* < 0.002). Box-and-whisker plot display sample mean (horizontal bar) and interquartile range from first to third quartiles (shadowed area). Whiskers indicate adjacent values (defined as ±1.5xIQR).

The length of hospital stay was longer in SD than ISEE (ISEE: 22 [15, 30] d vs. SD: 38 [29, 53] d, median [interquartile range], *p* < 0.001) as well as the length of ICU stay (ISEE: 0 [0, 4] d vs. SD: 3 [0, 12] d, *p* < 0.002) (Figure 5).

### 3.5. Occurrence rates of SD and ISEE in our centre between 2002 and 2021

**Figure 6:**
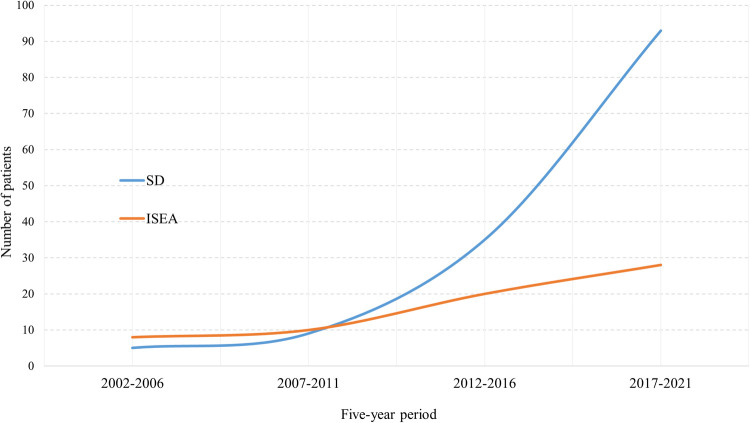
Five-year occurrence rates of SD and ISEE. This figure demonstrates the 5-year occurrence rate of spondylodiscitis (SD) and isolated spinal epidural empyema (ISEE) between 2002 and 2021 at our centre.

We observed a constantly increasing number of SD and ISEE cases over the last 20 years in our centre. SD cases occurred in a significantly higher range than ISEE cases (*p* < 0.001). We identified 13 cases between 2002 and 2006 (ISEE: 8 vs. SD: 5, *p* = 0.581), 19 cases between 2007 and 2011 (ISEE: 10 vs. SD: 9, *p* = 1.0), 55 cases between 2012 and 2016 (ISEE: 20 vs. SD: 35, *p* = 0.059), and 121 cases between 2017 and 2021 (ISEE: 28 vs. SD: 93, *p* < 0.001) (Table 5 and Figure 6).

**Table 5:** Five-year occurrence rates of SD and ISEE at our center between 2002 and 2021.

Five-year period	SD	ISEE	SD & ISEE	*p*-value
2002–2006	5	8	13	0.581^*^
	38.5%	61.5%	100%	
2007–2011	9	10	19	1.0^*^
	47.4%	52.6%	100%	
2012–2016	35	20	55	0.059^*^
	63.6%	36.4%	100%	
2017–2021	93	28	121	**<0**.001^*^
	76.9%	23.1%	100%	
total	142	66	208	**<0**.001^**^
	68.3%	31.7%	100%	

SD, spondylodiscitis; ISEE, isolated spinal epidural empyema.

*Binomial test.

**Chi-squared test.

Bold values are significant results (*p* < 0.05) as indicated in the methods.

### 3.6. Risk factor associations of complications

Univariate and multivariate analyses are summarized in (Table 6). Sex (*p* = 0.036; OR: 0.430; 95% CI: 0.195–0.948) and entities (*p* < 0.001; OR: 0.151; 95% CI: 0.066–0.346) were identified as independent risk factors for development of complications such as sepsis, septic embolism, endocarditis, relapse, and disease-related mortality. Univariate analysis revealed that sex, age over 65 years, and entities were associated with complications, relapse, and disease-related mortality.

**Table 6:** Univariate and multivariate logistic regression analysis to identify independent risk factors for the development of complications.

Variables	Univariate logistic regression		Multivariate logistic regression	
	OR (95% CI)	*p*-value	OR (95% CI)	*p*-value
Age >65 years	2.245 (1.160–4.345)	**0**.**016**	1.817 (0.850–3.886)	0.124
Sex	0.476 (0.243–0.934)	**0**.**031**	0.430 (0.195–0.948)	**0**.**036**
Diabetes mellitus	0.647 (0.325–1.287)	0.215	0.861 (0.394–1.882)	0.707
Obesity (BMI > 30 kg/m^2^)	0.909 (0.455–1.816)	0.787	1.677 (0.710–3.960)	0.238
Immunocompromised/Immunosuppressed	0.767 (0.320–1.833)	0.550	0.922 (0.350–2.429)	0.870
Entities (SD/ISEE)	0.136 (0.064–0.288)	**<0**.**001**	0.151 (0.066–0.346)	**<0**.**001**

SD, spondylodiscitis; ISEE, isolated spinal epidural empyema, BMI, body mass index, OR, odds ratio, CI, confidence interval. Complications noted include sepsis, septic embolism, endocarditis, relapse, and disease-related mortality.

Bold values are significant results (*p* < 0.05) as indicated in the methods.

## 4. Discussion

Our study provides insights into the management and clinical course of SD and ISEE and identifies distinct entity-related patterns of the characteristics and outcomes. In previous studies, SD and ISEE were diagnosed in male patients twice as often as females (22–25). In our cohort, SD occurred predominantly in male patients and was associated with a significantly higher age at diagnosis than patients in the ISEE group, in which the gender distribution was more balanced.

Diabetes mellitus, obesity, immunosuppression, age, and gender are the most known risk factors (12). SD showed significantly more obese elderly male patients than SD. Patients with diabetes mellitus and immunosuppression were equally distributed in both groups.

The pathogen detection rate in our study is higher than in previous reports (26–28). We detected pathogens in 92.3% using blood cultures, intraoperative specimens, and/or CT-guided biopsies, with MSSA as the most common pathogen. MSSA is the predominant bacterial aetiology in ISEE and SD (5, 23, 24, 27, 29–38).

Current studies demonstrate that the lumbar spine is the most involved region in ISEE and SD patients, followed by the thoracic spine (12, 16, 17, 23, 38–42). 67.6% of our SD and 62.1% of ISEE cases occurred in the lumbar spine, while the cervical spine was the least affected.

It is difficult to determine whether an infected organ is the primary infectious source causing secondary bacteraemia or if the organ was infected secondarily due to a primary bacteraemia. Indeed, from a therapeutic perspective, this differentiation is less relevant since both the infected focus and the bacteraemia must be simultaneously treated (43). A primary infectious source can be identified in 60% of ISEE cases and in 50%–68% of SD cases (23, 44). We found a primary infectious source in 71.1% of SD cases and in 66.7% of ISEE cases.

Skin infections were reported to present the most common focus in patients with SD (32, 34, 35). Similarly, Krishnamohan et al. reported that skin infections were the most frequent infectious source for ISEE. This is concurrent with our study, in which skin infections demonstrated the major primary source in the SD and ISEE groups. Chae et al. identified in 26% of ISEE cases epidural infiltration or acupuncture as the infectious source, which is consistent with our study in ISEE patients (34.1%) (44, 45).

Consistent with previous studies, we treated our ISEE patients almost exclusively with abscess evacuation with/without drainage (46, 47). SD patients were mainly instrumented (ventral, dorsal, or ventrodorsal) which has been shown to be an effective procedure (23, 48).

A paraspinal abscess was found in approximately 47% of SD cases with/without epidural empyema (26, 27, 35, 49, 50). However, Chae, H. J. et al. found a psoas abscess in 65% of ISEE patients (45). A paraspinal or psoas abscess was identified in 61.5% of our cases (SD and ISEE combined), and CT-guided biopsy with abscess drainage was performed in 33.7% of cases. The occurrence of psoas or paraspinal abscess was higher in the SD than in our ISEE cohort (66.9% vs. 50%).

Our medical management includes initially empiric or targeted antibiotic therapy, depending on the clinical severity of the disease, according to the recommendations of the literature and guidelines of the Infectious Diseases Society of America (IDSA) with an individually adapted therapy (12, 31, 38, 51). Two weeks of intravenous and four weeks of oral antibiotic therapy were performed in the ISEE patients, while the SD patients received four weeks of intravenous and 6 weeks of oral antibiotics.

Complications are common in SD and ISEE patients. Severe sepsis with multiple organ failures in SD patients with/without epidural empyema were reported in 14%–20% of cases (36, 52). Similarly, approximately 18% of SD patients with/without epidural empyema had a septic embolism during the course of the disease (52). We found that SD patients were significantly more likely to develop sepsis or septic embolism during the disease course than their ISEE counterparts. The pathogenesis of spondylodiscitis is thought to be septic embolic spread in the endplates of the vertebral body, where the vessels terminate, due to bacteraemia. This may explain the frequency of further septic embolism compared to local infection as in ISEE.

Endocarditis in SD patients with/without epidural empyema was widely described, for instance Patzakis et al. reported on 2% of endocarditis cases in his cohort (53), Pojskic et al. 4.2% (36), Nolla et al. 6% (27), Chae et al. 9% (45), Chelsom et al. 10% (54), Geisler et al. 12% (52), Mylona also et al. 12% (23), Osenbach et al. 15% (34), Ledermann et al. 24% (55), and Pigrau et al. 31% (35). Endocarditis was observed significantly more frequent in the SD than in the ISEE group (18.4% vs. 1.9%), which may be due to the haematogenous dissemination of SD.

Clinical, microbiologic, and radiological relapse rates following completed antibiotic treatment has been used in numerous studies to evaluate the success of SI treatment (56–58). Relapse rate in SD and ISEE patients was reported in 8%–15% (36, 59, 60). We noted a higher rate of relapses in the SD group than in the ISEE group (29.3% vs. 8.7%). Mylona et al. reported a mortality rate of 6% in a systematic review of 14 previous studies (23), which was comparable to the mortality rate in our study. However, the mortality rate was higher in SD patients than in ISEE patients.

Patients with SD or ISEE spent an average of 31.5–34 days in the hospital (41, 61). We found that SD patients stayed significantly longer in the hospital compared with ISEE patients (38 vs. 22 days). In addition, SD and ISEE patients stayed in the ICU for an average of 4 days (±11.0, range 1–68) (61). Thus, SD patients in our cohort spent more time in the ICU than ISEE patients (median: 3 vs. 0 days).

The incidences of SD and ISEE in western countries are estimated between 0.2–2.4/100,000 and 2.5–3.0/100,000, respectively (14, 40, 62, 63), with an increasing incidence in the last decades (7, 16, 24, 30, 37, 39, 64). Indeed, at our institution we observed a significant increase in SD and ISEE cases in the last 20 years. However, SD was more common than ISEE (68.3% vs. 31.7%).

### 4.1. Limitations and strengths of this study

The limitations of our retrospective study include a possible selection bias toward more severe cases because of the high degree of specialization of our university centre. The single-centric nature of our analysis might reduce the external validity of our observations. However, our cohort analysis is based at 20-year period of SD treatment in a large university neurosurgery centre, suggesting high internal validity of our study. Therefore, our observations might be useful to understand the clinical outcomes in patients with ISEE and SD. Furthermore, our data provide a basis for the design of prospective observational and interventional research.

## 5. Conclusions

Patients with ISEE show a more favourable disease course (sepsis, septic embolism, endocarditis, relapse rate, disease-related mortality, and LOS in hospital/ICU) than SD patients. Gender, age, occurrence rate of disease over the last 20 years, primary infection source, causative pathogens, localization, type of surgical procedure, and duration of antibiotic therapy differ between both pathologies. Therefore, it appears probable that both pathologies constitute two separate entities with different requirements on clinical management. The differences between both entities warrant prospective, multicentre follow-up research to further elucidate clinical and pathophysiologic patterns in these diseases.

## Data Availability

The original contributions presented in the study are included in the article, further inquiries can be directed to the corresponding author.
